# Immunological Responses and Epitope Mapping by Tuberculosis-Associated Antigens within the RD1 Region in Japanese Patients

**DOI:** 10.1155/2014/764028

**Published:** 2014-01-28

**Authors:** Hideaki Nagai, Maho Suzukawa, Yumi Sakakibara, Ken Ohta, Pedro A. Reche, Koichi Suzuki, Yoshihiko Hoshino

**Affiliations:** ^1^National Hospital Organization, Tokyo National Hospital, 3-1-1 Takeoka, Kiyose, Tokyo 204-8585, Japan; ^2^Leprosy Research Center, National Institute of Infectious Diseases, 4-2-1 Aoba, Higashi-Murayama, Tokyo 189-0002, Japan; ^3^Facultad de Medicina, Department of Microbiology I-Immunology, Universidad Complutense de Madrid, 28040 Madrid, Spain

## Abstract

Tuberculosis remains a major global health problem worldwide, and hence there is a need for novel vaccines that better induce cellular-mediated immunity (CMI). In search of a better vaccine target, the QuantiFERON-TB Gold In-Tube Test (QFT-GIT) and the interferon-*γ* ELISPOT assay (ELISPOT) were used to compare the magnitude of CMI in patients. Results of the ELISPOT assay led to the discovery of specific epitopes within the early secreted antigenic target 6 kDa (ESAT-6) and culture filtrate protein 10 kDa (CFP-10) proteins. Both peptides showed a strong association with several HLA class II DRB1 molecules in the Japanese population. Using ESAT-6-specific HLA class II tetramers, we determined that the expression of ESAT-6-specific CD4+ lymphocytes was significantly decreased in treated patients compared with active patients. In addition, programmed death-1 (PD-1)/killer cell lectin-like receptor G1 (KLRG-1) double positive cells were found only in treated patients and not in those with active TB. These data could provide clues for the development of novel tuberculosis vaccines.

## 1. Introduction

Tuberculosis (TB), caused by *Mycobacterium tuberculosis* (*M. tuberculosis *(Mtb)), is still a major health problem around the world. It is second only to AIDS as the leading cause of death from infectious diseases. The World Health Organization estimates that there were 9 million new cases of TB and 1.4 million TB-related deaths in 2011 [1].

The effectiveness of the only available TB vaccine, Bacillus Calmette-Guérin (BCG), is limited. Protection in children has only been demonstrated for disseminated TB and tuberculosis meningitis [2], while the efficacy for adult pulmonary TB is very variable [1]. In fact, a controlled trial of BCG failed to demonstrate its effectiveness in protecting against the development of TB [3]. Lack of efficacy of BCG for adult TB is a serious problem in controlling the disease and may be partly related to the fact that the efficacy of BCG vaccinations declines over time. The results of several epidemiological studies suggest that the effectiveness of BCG vaccinations lasts for around twenty years [4, 5]. This decline of efficacy would be associated with a decrease of cell-mediated immunity (CMI) to Mtb. An assessment of the degree of CMI over time would require objective, repeatable, and economical assays.

The tuberculin skin test (TST), an assay for type IV-delayed hypersensitivity reaction, is the primary screening method used to determine TB exposure. The presence or absence of induration following the TST injection can be used as objective proof of active CMI against mycobacterial infection [6]. However, the TST cannot distinguish Mtb from BCG or nontuberculous mycobacteria (NTM) [7]. Therefore, an *in vitro* (or *ex vivo*) interferon-*γ* (IFN-*γ*) release assay (IGRA), which utilizes antigens within the region of difference 1 (RD1) of *M. tuberculosis*, is used in conjunction with the TST. These antigens, such as early secreted antigenic target 6 kDa (ESAT-6) (Rv3875), culture filtrate protein 10 kDa (CFP-10) (Rv3874), and TB7.7 (Rv2654c), are not present in BCG or most environmental NTM [8]. These antigens are also very potent inducers of CMI, even in an *in vitro *setting [9, 10], which enables the establishment of an ELISA-based assay system to measure IFN-*γ* secreted into the culture supernatant after antigenic stimulation.

QuantiFERON-TB Gold (QFT-G) is an IGRA that utilizes two Mtb-specific antigens, ESAT-6 and CFP-10. Its successor, the QuantiFERON-TB Gold In-Tube Test (QFT-GIT), incorporated TB7.7 in addition to ESAT-6 and CFP-10 [11]. A simultaneous and longitudinal comparison of the QFT-G and QFT-GIT assays among healthcare workers showed that QFT-GIT is the more sensitive of the two [12], probably due to the addition of TB7.7 [13].

Another IGRA, T-SPOT.TB, uses an ELISPOT assay to measure IFN-*γ* [11]. However, T-SPOT.TB uses only two antigens, ESAT-6 and CFP-10, to stimulate samples. Results from previous methods were significantly affected by the lymphocyte count in the peripheral blood of each patient [15]. Therefore, we aimed to analyze precise epitopes of Mtb-specific antigens in CD4+ cells, which are the predominant cell type that secretes IFN-*γ* upon stimulation with Mtb antigens. We developed an ELISPOT assay (ELISPOT) using multiple, overlapping peptides from three proteins, ESAT-6, CFP-10, and TB7.7, to stimulate memory T cells.

In this study, the sensitivity of two IGRA assays, QFT-GIT and ELISPOT, was compared using blood samples from the same TB patients and the same tuberculosis antigen sets to determine the activity of CMI in patients. ELISPOT, using the same antigen peptide sets, was then used to analyze T cell response to Mtb-specific antigens to identify restricted epitopes. Finally, Mtb-specific CD4+ lymphocytes were segregated to evaluate their phenotype in TB patients.

## 2. Materials and Methods

### 2.1. Participants

Patients of Tokyo National Hospital in Tokyo, Japan, were consecutively enrolled in the study, after giving informed consent, from April 2010 to April 2011. A total of 177 Japanese patients (age: 58.5 ± 18.2 yr; male: 68.5%) were recruited. The following information was obtained from all patients at the time of enrollment: history of prior TB disease, work history in any healthcare settings or recent exposure to a patient with active TB, and other TB risk factors such as taking immunosuppressive drugs. Information on previous medical history, any clinical symptoms and signs, and radiological and microbiological data were also collected. The patients were then divided into three categories: (1) active disease: patients having positive symptom(s) and positive smear results and/or positive demonstration of Mtb in culture; (2) past disease: previously diagnosed with TB, treated, and currently free from symptom(s); and (3) latent TB infection (LTBI): no symptoms with normal chest X-ray but having positive results from an IGRA. Among the 177 patients recruited, 56 (32%) had active disease, 103 (58%) were in the past disease group, and 18 (10%) were classified as LTBI. For those patients classified as having the disease in the past, the average time period since disease onset was 2605 ± 605 (mean ± SE) days. The research protocol was approved by the Institutional Review Board of Tokyo National Hospital and by the Research Ethics Committee of the National Institute of Infectious Disease, Tokyo, Japan.

### 2.2. QuantiFERON-TB Gold In-Tube (QFT-GIT) Assay

The QFT-GIT assay was performed using fresh whole blood in accordance with the manufacturer's instructions (Cellestis, Chadstone, Australia). The results were interpreted with software provided by Cellestis. Results were scored as “positive” if the IFN-*γ* concentration in the tube with TB-specific antigen was >0.35 IU/mL after subtracting the value of the nil control and at least >25% of the negative control value. If the net IFN-*γ* response was <0.35 IU/mL for the antigens and the response to the mitogen-positive control was >0.5 IU/mL, the response was considered “negative” [15].

### 2.3. Peptides

Peptides 15 amino acids in length, with nine overlapping residues, were synthesized to cover the entire length of ESAT-6, CFP-10, and TB7.7. Some peptides were longer or shorter at the C- or N-terminus. The peptides were prepared by Dr. Imajoh-Ohmi in the Medical Proteomics Laboratory, Institute of Medical Science, University of Tokyo, Tokyo, Japan [16]. The purity of the peptides was >95% after purification with reversed-phase HPLC. The CFP-10 peptides were labeled as C1 to C16, ESAT-6 peptides as E1 to E15, and TB7.7 peptides as T1 to T13. Supplemental Table 1 (see Supplementary Material available online at http://dx.doi.org/10.1155/2014/764028) lists the sequence of each peptide.

### 2.4. HLA Typing

Peripheral blood mononuclear cells (PBMCs) were separated from heparinized blood samples by density centrifugation using BD Vacutainer Cell Preparation Tubes (Becton, Dickinson and Company, Franklin Lakes, NJ, USA). Genomic DNA was isolated from PBMCs with QIAamp DNA Blood Kits (Qiagen, Germantown, MD, USA) for HLA typing using the Luminex Multi-Analyte profiling system (xMAP; Luminex, Austin, TX, USA) [17].

### 2.5. ELISPOT Assay

The IFN-*γ* ELISPOT assay was performed to compare the sensitivity between IGRAs and to find an immunological epitope to tuberculosis-specific antigens. The peptide mixture of each protein was used to obtain sensitivity measurements, while a single peptide was applied to each well for epitope determination. PBMCs were seeded into precoated IFN-*γ* ELISPOT plates (Becton, Dickinson and Company, Franklin Lakes, NJ, USA) with 2.5 × 10^5^ cells per well in AIM-V medium (GIBCO) and incubated with one of a series of peptides (10 *μ*M) or a peptide mixture (10 *μ*M) of each antigen at 37°C in 5% CO_2_ for 16 h. A negative control (no mitogen or antigen) and a positive control (phytohemagglutinin, PHA, 5 g/mL) were also included. After incubation, the wells were washed and developed with a conjugate against the antibody used and an enzyme substrate. Spot-forming units were counted using a KS ELISPOT imaging system (Carl Zeiss, Hallbergmoos, Germany) as spot-forming cells (SFC). ELISPOT results were interpreted according to the following criteria: the test result was positive when (1) the negative control had 0–5 spots and (2) the (antigen spot count) − (negative control spot count) was greater than six. The test result was negative if the above criteria were not met and the positive control was valid (≥20) [18].

### 2.6. Tetramer Staining and Flow Cytometric Analysis

HLA class II tetramers conjugated with APC-labeled streptavidin were provided by the tetramer core laboratory at the National Institutes of Health, Bethesda, MD, USA. PBMCs were incubated with class II tetramers for 2 h at room temperature. The following fluorescence-labeled monoclonal antibodies (mAbs) were used in this study: anti-CD3-APCCy7 (HIT3a), anti-CD8-PerCP-Cy5.5 (MAb11) (Biolegend, San Diego, CA, USA), and anti-CD4-Pacific Blue (OKT4) (eBioscience, San Diego, CA, USA), anti-PD-1-PE (BD Bioscience). Anti-KLRG-1-Alexa488 was kindly provided by Professor H. Pircher (University of Freiberg, Germany) [19]. Where necessary, the relevant isotype control mAb was used. Cell viability was assessed using the LIVE/DEAD Kit (Invitrogen, Carlsbad, CA, USA). Following a 30 min incubation at 4°C, the cells were washed and acquired using a FACS Canto II flow cytometer (BD Bioscience) [20]. FACS data were reanalyzed using FlowJo software, version 8.8.7 (TreeStar, San Carlos, CA, USA).

### 2.7. Statistical Analysis

Group medians and distributions were analyzed using the Wilcoxon matched-pairs signed-rank test and the Mann-Whitney *U* test. All analyses were performed using GraphPad Prism software, version 5 (San Diego, CA, USA). The threshold of significance was set at *P* < 0.05.

## 3. Results

### 3.1. Comparison of Two IGRAs and the Identification of Antigenic Peptides

**Figure 1:**
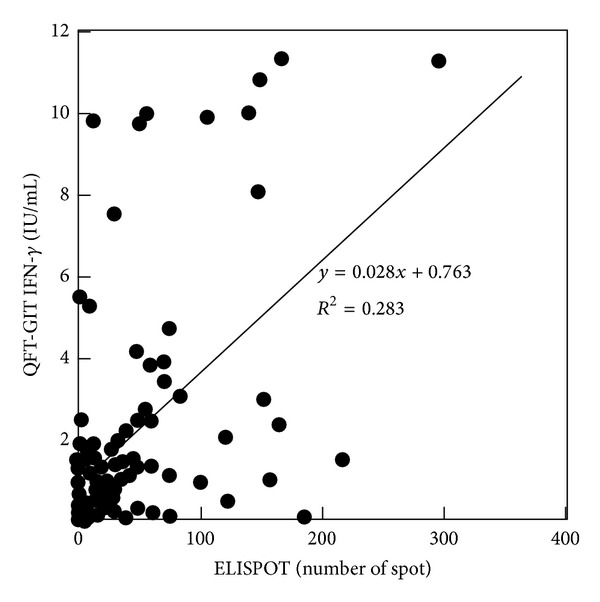
Association between QFT-GIT values and number of spots in the ELISPOT assay (*r* = 0.532; *P* < 0.001).

All patient samples (*n* = 177) were analyzed by QFT-GIT and 115 were analyzed by ELLISPOT. As expected, when the results from both assays were compared, there was a positive correlation between QFT-GIT values and the number of spots obtained in the ELISPOT assay (*r* = 0.532; *P* < 0.001) (Figure 1). The detection rate of each assay was compared across the three groups of patients: active disease, past disease, and LTBI. QFT-GIT failed to detect many patients with active and past disease; ELISPOT detection was more consistent (overall detection rate of 93.9% versus 65.5%) (Table 1). The average duration from disease onset to the date of the assay was 2, 086 ± 743 days in QFT-GIT-negative past TB patients and 4, 920 ± 3042 days in ELISPOT-negative past TB patients. When both assays were compared in the same patients, 31 of 37 QFT-GIT-negative cases (84%) were positive by ELISPOT (Table 2), suggesting that the ELISPOT assay developed in our laboratory was better at detecting a broader range of TB+ patient populations. Consequently, the ELISPOT was chosen to carry out the detection of antigenic peptides in Mtb-specific proteins.

Each Mtb-specific antigen used in the IGRA comparison was further evaluated to identify the peptide(s) that most efficiently induced antigenicity to Mtb and activation of host CMI. We found that 77% of patients responded to ESAT-6 peptides and 66% to CFP-10, while no single case responded to TB7.7 (data not shown). TB7.7 peptides were excluded from further consideration in the study.

### 3.2. Identification of Specific ESAT-6 and CFP-10 Epitopes

ELISPOT data provided an indication of the prevalence and strength of the ESAT-6 and CFP-10 Mtb antigens. The next step was to map the precise epitope(s) of the Mtb-specific antigens recognized by CD4+ lymphocytes. Two sets of peptides were synthesized, 15 overlapping ESTA-6 peptides (E1–E15) and 16 overlapping CFP-10 peptides (C1–C16) (Supplemental Table 1), to evaluate patient samples using ELISPOT.

**Figure 2:**
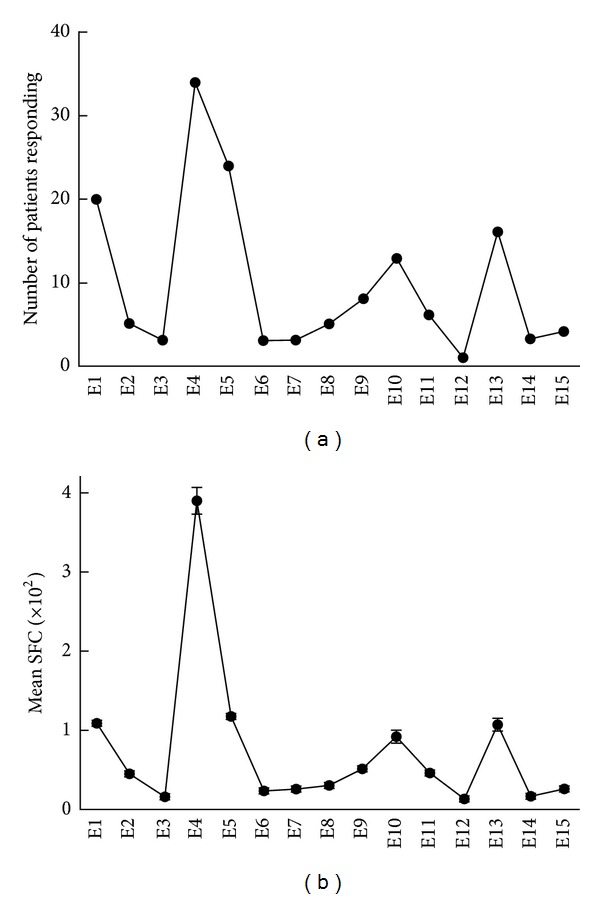
Antigenic regions from ESAT-6. PBMCs were incubated with overlapping peptides from ESAT-6 (E1–E15), and the number of IFN-*γ* producing cells was measured using the ELISPOT assay. Shown are numbers of donors who responded (a) and the mean (±SE) number of SFCs (spot forming cells)/10^7^ PBMCs in response to each peptide (b).

**Figure 3:**
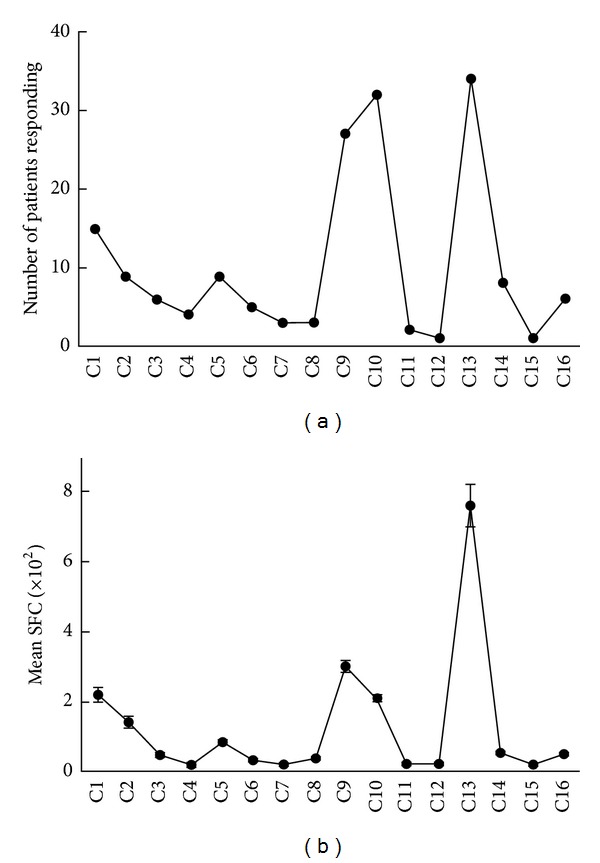
Antigenic regions from CFP-10. PBMCs were incubated with overlapping peptides from CFP-10 (C1–C16), and the number of IFN-*γ* producing cells was measured using the ELISPOT assay. Shown are numbers of donors who responded (a) and the mean (±SE) number of SFCs (spot forming cells)/10^7^ PBMCs in response to each peptide (b).

Among the 15 peptides from ESTA-6, E1, E4, E5, E10, and E13 elicited a response in multiple patients (Figure 2(a)). The same peptides induced a higher average number of spot-forming cells (SFC) in the ELISPOT assay (Figure 2(b)), suggesting that these are major epitopes that stimulate CD4+ cells in peripheral blood. Similarly, peptides C1, C5, C9, C10, and C13 from CFP-10 were identified as responsible epitopes that stimulated multiple patients (Figure 3(a)) to induce a stronger IFN-*γ* response (Figure 3(b)).

### 3.3. Identification of an HLA DRB1 Haplotype Unique to TB Patients

Although CMI to TB involves both CD4+ and CD8+ positive lymphocytes [21, 22], the primary source of IFN-*γ* as measured by ELISPOT is CD4+ cells [14, 23]. CD4+ T lymphocytes are activated by specific antigens presented by antigen presenting cells (APCs) through major histocompatibility complex (MHC) class II molecules (or HLA-DR in humans). Therefore, to ascertain specificities to each ESAT-6 and CFP-10 peptide, human HLA DRB1 haplotypes were examined by employing a high-resolution Luminex-based method.

As shown in Table 3, the proportion of DRB1 identified in TB patients was most similar to that found in the Japanese population (*n* = 916). The predominant haplotype was DRB1*0405 (13.5%), followed by DRB1*0901 (12.9%), DRB1*1502 (12.6%), and DRB1*0803 (8.0%). However, some haplotypes were significantly higher or lower than those in the Japanese control population. They included DRB1*0101 (1.8% in TB versus 4.8% in control), DRB1*0406 (7.1% versus 3.2%), and DRB1*1502 (12.6% versus 8.7%).

### 3.4. Association between HLA Class II and the Epitopes

**Figure 4:**
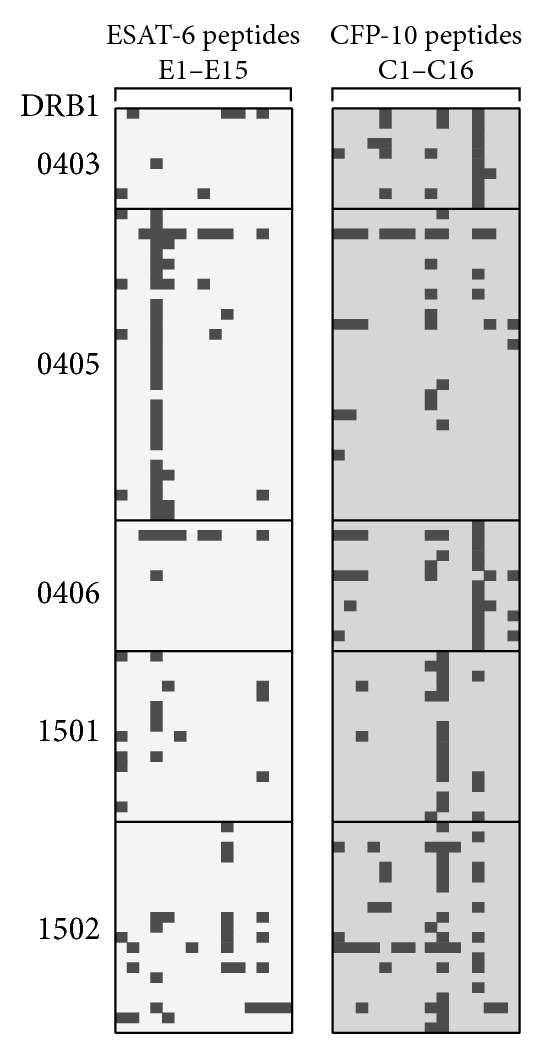
Association between HLA class II DRB1 and epitopes. PBMCs were incubated with a single overlapping peptide from CFP-10 (C1–C16) or ESAT-6 (E1–E15). The positive well was labeled.

The ability of each ESAT-6 and CFP-10 peptide to induce IFN-*γ* in ELISPOT was examined and its relation to each HLA DRB1 haplotype was analyzed. Haplotypes that showed characteristic results are illustrated in Figure 4, in which positive spots as described in Section 2.5 are shown in dark grey. Among the ESAT-6 peptides identified in the previous analysis (Figures 2 and 3), E4 showed a strong association with DRB1*0405 (28 out of 31 cases) and weak associations with DRB1*1501 (5 out of 17 cases) and DRB1*1502 (4 out of 21 cases) (Figure 4, left panel). Likewise, the C10 peptide from CFP-10 showed strong associations with DRB1*1501 (17 cases) and DRB1*1502 (21 cases).

Peptide-MHC binding is the most selective of the events that determine T cell epitopes. Thus, peptide-MHC binding motif profiles can be used to predict the identity of T cell epitopes [24–26]. We predicted HLA DRB1*0405-restricted T cell epitopes using *in silico* analysis of peptide-MHC binding profiles derived from peptides known to bind the relevant MHC molecules [24–26]. A given peptide bound to a specific HLA molecule was considered a potential CD4+ T cell epitope when its binding score ranked within the top 3% percentile of scores obtained for 1000 random 9-mer peptides (average amino acid composition of proteins in the SwissProt database), using the same profile. Peptide-MHC class II binding profiles only predict the 9-mer core that fits in the binding groove [24]. However, we also provided the three most proximal N-terminal and C-terminal residues, as they can also be the target of T cell recognition [24]. As a result, the minimal core region of the E4 peptide is postulated as QGN**VTSIHSLLD**EGK (the bold portion is the minimal core region).

### 3.5. Evaluation of ESAT-6-Specific CD4+ Lymphocytes in Active and Past TB Patients

Although ELISPOT is a highly sensitive method for the identification of TB patients, it is still difficult to differentiate active patients from past patients using IGRAs. Another solution was needed to distinguish between the two. Based on the epitope information described above, an ESAT-6-specific tetramer (QGNVTSIHSLLDEGK) was synthesized with HLA DRB1*0405. Another tetramer (PVSKMRMATPLLMQA) provided by the NIH tetramer facility was used as the negative control. The PMBCs of one active and one past TB patient were stained with DRB1*0405 ESAT-6 tetramer using a modification of a previous method [20].

**Figure 5:**
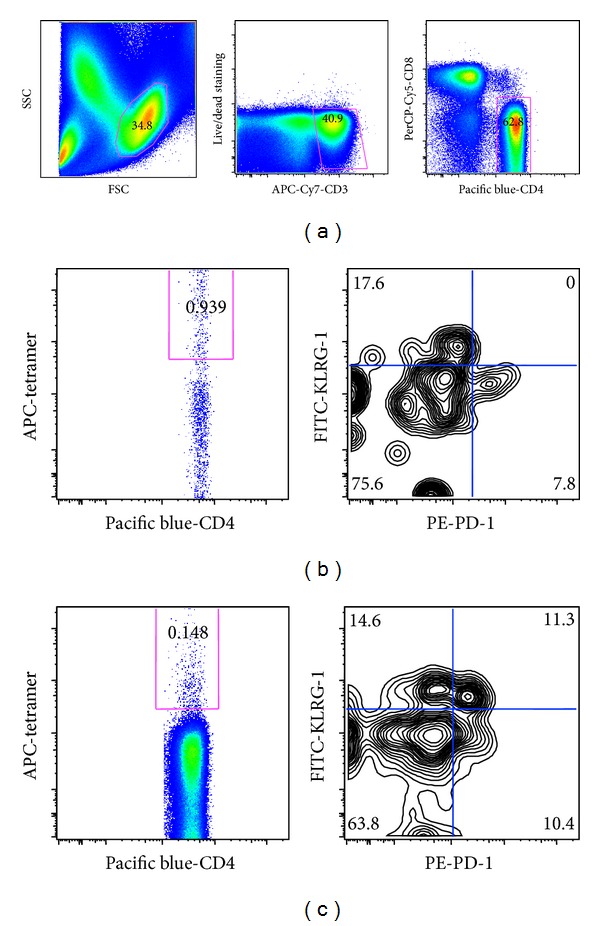
ESAT-6-specific CD4+ lymphocytes in active TB patients (b) and past TB patients (c) stained with MHC class II tetramers. Plots are gated on lymphocytes (by FSC and SSC), live CD3+ cells (by live/dead staining and CD3), and CD4+ cells (by CD4 and CD8) (a). The gated CD4+ lymphocytes are gated with MHC class II tetramer and KLRG-1 and PD-1.

PBMCs were gated with SSC and FSC to isolate the lymphocyte fraction after deletion of doublets using FSC-A and FSC-H. Lymphocytes were stained with anti-CD3, CD4, CD8, and live/dead dye to eliminate dead CD4+ T cells (Figure 5(a)). Viable CD4+ lymphocytes were then gated with ESAT-6-specific tetramer (Figures 5(b) and 5(c), left panels). A lower proportion of ESAT-6-specific CD4+ lymphocytes was found in the past TB patient than in the active TB patient (0.148% versus 0.939%, Figures 5(b) and 5(c), left panels). Antiprogrammed death-1 (PD-1) and antikiller cell lectin-like receptor G1 (KLRG-1) were then used to distinguish the proliferation and cytokine production phenotypes of the CD4+ cells [27]. PD-1 expressing CD4+ lymphocytes possess proliferative capacity, while KLRG-1 expressing CD4+ lymphocytes are relatively short lived but have cytokine secretion capacity. Although PD-1 positive and KLRG-1 positive CD4+ T cells were identified in both cases, PD-1 and KLRG-1 double positive CD4+ lymphocytes were detected only in the past TB patient (Figure 5(c) versus Figure 5(b), right panels).

## 4. Discussion

In this study, we first compared the ability of two IGRAs to detect TB infections in order to evaluate CMI to TB. Both assays were mutually acceptable for this purpose, but the ELISPOT was more sensitive than QFT-GIT. The ELISPOT was then used to determine antigenic dominant regions in Mtb-specific proteins. Several epitopes of Mtb-specific antigens were found in CD4+ lymphocytes. Using these epitopes, we made an ESAT-6-specific MHC class II tetramer to detect the kinetics of tuberculosis-specific CD4+ lymphocytes. The proportion of ESAT-6-specific CD4+ lymphocytes was significantly reduced following treatment.

There are many studies comparing QFT-GIT and ELISPOT, especially for individuals suspected of having tuberculosis or a latent TB infection [28–30]. In most cases, ELISPOT or the commercially available T-SPOT.TB was more sensitive than QFT-GIT with a similar specificity. Moreover, in this study, we were able to detect specific epitopes of the Mtb-specific antigens.

Three different patient populations were used for several reasons. We first used active TB patients. A time lapse between bacterial growth and appearance of the adaptive immune response has been observed in mice as well as in humans [31]. Around 30% of active patients were negative by QFT-GIT, suggesting that early evaluation of TB might lead to misdiagnosis. Next, past TB patients were recruited to investigate the continuation of the host immune response to Mtb. QFT-GIT and ELISPOT exhibited differences in time course when the IGRA results were negative (5 yr versus 13 yr). As expected, ELISPOT was much more sensitive and became negative afterwards. However, the age factor could not be adjusted because of the small population size. Lastly, we collected LTBI cases. However, the numbers were too small for meaningful analysis of any differences. Indeed, among 18 LTBI cases, 4 were diagnosed by QFT-GIT only and 6 by ELISPOT; only 8 cases were diagnosed by both.

With the ELISPOT assay established in our laboratory, we used overlapping peptides from antigenic proteins, not an empirical mixture of peptides, to detect distinct antigenic regions in ESAT-6 and CFP-10. Arlehamn et al. identified similar regions; however, as the ethnicity of the two groups of subjects was considerably different, the regions were not expected to be identical [14]. This is true of the distribution of MHC class II. DRB1*0405 is the most prevalent in the Japanese population and, as expected, the most frequent population in TB patients.

None of the patients responded to the TB7.7 peptides, which was consistent with the results of a US study [14]. This was surprising, given that this antigen is a recent addition to QFT-GIT tests. This finding suggests that the increased sensitivity of QFT-GIT tests is due to stimulation by the mixture of antigens in the same tube rather than addition of the novel TB7.7 antigen.

PD-1 and KLRG-1 were used as phenotypic markers for ESAT-6-specific CD4+ lymphocytes. In the LCMV model, PD-1 expression on CD8+ lymphocytes is a marker of cell exhaustion; however, in the tuberculosis model, CD4+ lymphocytes that express PD-1 have proliferative potential, suggesting that these are effector CD4 cells [27]. In contrast, KLRG-1 expression is associated with terminal differentiation and induces cytokine secretion. Reiley et al. examined several other activation markers such as CD44, CD62L, CD27, and CD127 (IL-7R*α*) and found that they do not differ between the KLRG-1- and PD-1- expressing cell populations during tuberculosis infection [27]. Our results suggest that, in the active phase, ESAT-6-specific CD4+ lymphocytes, expressing either PD-1 or KLRG-1, are dominant. However, in the chronic phase, PD-1 expression might decline and KLRG-1+ or PD-1+/KLRG-1+ CD4+ lymphocytes are dominant, although a recent mouse study showed that a significant portion of ESAT-6-specific PD-1 expressing CD4+ lymphocytes also express KLRG-1. The primary limitation of this study was the small sample size. Further precise study with human peripheral blood is required with a larger population of participants [32].

## 5. Conclusion

The ELISPOT assay was more sensitive than QFT-GIT in evaluating the adaptive immunity to TB. In addition, the use of overlapping peptides revealed the association between epitopes of two Mtb peptides in CD4+ lymphocytes and MHC class II haplotypes. Finally, a significant difference in the expression of ESAT-6-specific CD4+ lymphocytes was discovered based on stage of treatment.

## Supplementary Material

Supplemental Table:
Peptide sequences of tuberculosis-specific antigens. CFP-10 is a 100 amino acid (a.a.), ESAT-6 has 95 a.a., and TB7.7 has 81 a.a..

## Figures and Tables

**Table 1:** Sensitivity of IGRAs. Patients were divided into three categories: active disease, past disease, and latent TB infection (LTBI). QFT-GIT was conducted in 177 patients and ELISPOT in 115 patients.

	QFT-GIT (*n* = 177)		ELLISPOT (*n* = 115)	
	(−)	(+)	(−)	(+)
Active disease	17 (9.6%)*	39 (22.0%)	1 (0.9%)**	35 (30.4%)
Past disease	38 (21.5%)	65 (36.7%)	6 (5.2%)	59 (51.3%)
LTBI	6 (3.4%)	12 (6.8%)	0 (0%)	14 (12.1%)
Total	61 (34.5%)	116 (65.5%)	7 (6.1%)	108 (93.9%)

*Percentage of total number tested using QFT-GIT (*n* = 177).

**Percentage of total number tested using ELISPOT (*n* = 115).

**Table 2:** Correspondence between two IGRAs.

	ELLISPOT (−)	ELLISPOT (+)	Total
QFT-GIT (−)	6	31	37
QFT-GIT (+)	1	77	78

Total	7	108	115

**Table 3:** Proportion of HLA class II DRB1 in Japanese TB patients and the general Japanese population. The asterisks show that the proportion of an HLA molecule in TB patients was significantly higher or lower than in the Japanese population.

DRB1	TB pts		Japanese population	
	Cases	Proportion	Cases	Proportion
0101*	6	1.8%	44	4.76%
0301	1	0.3%		
0401	7	2.1%	11	1.16%
0403	15	4.6%	27	3.00%
0404	2	0.6%	2	0.22%
0405	44	13.5%	142	15.51%
0406*	23	7.1%	29	3.22%
0410	7	2.1%	17	1.82%
0701	1	0.3%	7	0.81%
0802	9	2.8%	46	4.99%
0803	26	8.0%	69	7.55%
0901	42	12.9%	113	12.38%
1001	1	0.3%		
1101*	4	1.2%		
1201	20	6.1%	36	3.90%
1202	3	0.9%	24	2.66%
1301	3	0.9%	8	0.86%
1302	21	6.4%	48	5.25%
1401	7	2.1%	41	4.45%
1403	6	1.8%	14	1.57%
1405	4	1.2%	23	2.56%
1406*	3	0.9%		
1454	1	0.3%	0	0.00%
1501	25	7.7%	56	6.08%
1502*	41	12.6%	80	8.74%
1602	4	1.2%	5	0.55%

Total	326		916	
