# Supplementary material for: Immunological Responses and Epitope Mapping by Tuberculosis-Associated Antigens within the RD1 Region in Japanese Patients
Source: J Immunol Res. 2014 Jan 28;2014:764028. doi: 10.1155/2014/764028 (PMC3987935; doi:10.1155/2014/764028)
Supplement: Supplementary file 1 — Supplemental Table: Peptide sequences of tuberculosis-specific antigens. CFP-10 is a 100 amino acid (a.a.), ESAT-6 has 95 a.a., and TB7.7 has 81 a.a.. [file 764028.f1.pdf]

Supplemental Table 1. Peptide sequences of tuberculosis-specific antigens. CFP-10 is a 100 amino acid (a.a.) protein, ESAT-6 has 95 a.a., and TB7.7 has 81 a.a.

ESAT-6

MTEQQWNFAGIEAAASAIQGNVTSIHSLLDEGKQSLTKLAAAWGGSGSEAYQGVQQKWDATATELNNALQNLARTISEAGQAMASTEGNVTGMFA

E1	1:15	MTEQQWNFAGIEAAA
E2	7:21	NFAGIEAAASAIQGN
E3	13:27	AAASAIQGNVTSIHS
E4	19:33	QGNVTSIHSLLDEGK
E5	25:39	IHSLLDEGKQSLTKL
E6	31:45	EGKQSLTKLAAAWGG
E7	37:51	TKLAAAWGGSGSEAY
E8	43:57	WGGSGSEAYQGVQQK
E9	49:63	EAYQGVQQKWDATAT
E10	55:69	QQKWDATATELNNAL
E11	61:75	TATELNNALQNLART
E12	67:81	NALQNLARTISEAGQ
E13	73:87	ARTISEAGQAMASTE
E14	79:93	AGQAMASTEGNVTGM
E15	85:95	STEGNVTGMFA

CFP-10

MAEMKTDAATLAQEAGNFERISGDLKTQIDQVESTAGSLQGQWRGAAGTAAQAAVVRFQEAANKQKQELDEISTNIRQAGVQYSRADEEQQQALSSQMGF

C1	1:15	MAEMKTDAATLAQEA
C2	7:21	DAATLAQEAGNFERI
C3	13:27	QEAGNFERISGDLKT
C4	19:33	ERISGDLKTQIDQVE
C5	25:39	LKTQIDQVESTAGSL
C6	31:45	QVESTAGSLQGQWRG
C7	37:51	GSLQGQWRGAAGTAA
C8	43:57	WRGAAGTAAQAAVVR
C9	49:63	TAAQAAVVRFQEAAN
C10	55:69	VVRFQEAANKQKQEL
C11	61:75	AANKQKQELDEISTN
C12	67:81	QELDEISTNIRQAGV
C13	73:87	STNIRQAGVQYSRAD
C14	79:93	AGVQYSRADEEQQQA
C15	85:99	RADEEQQQALSSQMG
C16	91:100	QQALSSQMGF

TB7.7

MSGHALAARTLLAAADELVGGPPVEASAAALAGDAAGAWRTAAVELARALVRAVAESHGVAAVLFAATAAAAAAVDRGDPP

T1	1:15	MSGHALAARTLLAAA
T2	7:21	AARTLLAAADELVGG
T3	13:27	AAADELVGGPPVEAS
T4	19:33	VGGPPVEASAAALAG
T5	25:39	EASAAALAGDAAGAW
T6	31:45	LAGDAAGAWRTAAVE
T7	37:51	GAWRTAAVELARALV
T8	43:57	AVELARALVRAVAES
T9	49:63	ALVRAVAESHGVAAV
T10	55:69	AESHGVAAVLFAATA
T11	61:75	AAVLFAATAAAAAAV
T12	67:81	ATAAAAAAVDRGDPP
T13	73:81	AAVDRGDPP
